# The first presentation of Wolcott‐Rallison syndrome in a four‐month‐old infant with diabetic ketoacidosis (DKA) precipitating by COVID‐19: A case report

**DOI:** 10.1002/ccr3.5096

**Published:** 2021-11-19

**Authors:** Elham Maleki, Amir Baniasad, Mina Sepehran, Najmeh Davoudian

**Affiliations:** ^1^ Endocrinology and Metabolism Research Center Institute of Basic and Clinical Physiology Science Kerman University of Medical Sciences Kerman Iran; ^2^ Infectious Diseases Research Centre Gonabad University of Medical Sciences Gonabad Iran

**Keywords:** coronavirus disease 2019, diabetic ketoacidosis, hyperglycemia, neonatal diabetes, Wolcott‐Rallison syndrome

## Abstract

Monogenic diabetes mellitus (eg, Wolcott‐Rallison syndrome) is a rare condition. It associates with neonatal or early‐infancy insulin‐dependent diabetes. We reported DKA in the four‐month infant as the first presentation of monogenic diabetes that has accelerated by COVID‐19 infection. Therefore, considering the concurrency of COVID‐19 and DKA is crucial.

## Graphical abstract

DKA as an early manifestation of monogenic diabetes (eg, Wolcott‐Rallison syndrome) in infancy, can be accelerated by COVID‐19 infection, so its distinction is imperative in clinical settings.

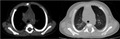

## 1 INTRODUCTION

Wolcott‐Rallison syndrome (WRS) (OMIM 226980) is a rare autosomal recessive disease that for the first time was reported in 1972.^1^ It is the most common genetic cause of permanent neonatal diabetes mellitus and is more common in consanguineous families.^2^ Other manifestations of the WRS, including skeletal dysplasia, osteoporosis, acute liver failure, renal impairment, neutropenia, recurrent infections, hypothyroidism, intellectual deficit, and exocrine pancreas insufficiency, commonly occur at older ages.^3^, ^4^ Previous studies have shown the homozygous mutations in the Eukaryotic Translation Initiation Factor 2‐ALPHA Kinase 3 (EIF2AK3) gene cause WRS.^5^

In late 2019, a new coronavirus has identified as the cause of pneumonia in China. It has spread rapidly and has become a pandemic worldwide.^6^ Coronavirus disease 2019 (COVID‐19) can affect any age group and has different clinical manifestations. Pneumonia seems to be the most common and severe manifestation of COVID‐19 infection. It is characterized by fever, coughs, and dyspnea, but asymptomatic infections have also been reported.^7^, ^8^

COVID‐19 is caused by severe acute respiratory syndrome coronavirus 2 (SARS‐CoV‐2) and it enters the cells by binding to ACE2 receptors. These receptors are present in the body's major organs and tissues, including lung, pancreatic cells, adipose tissue, small intestine, and kidneys.^9^ The virus may lead to acute dysfunction of pancreatic beta cells, leading to acute hyperglycemia.^10^

This study aimed to report Wolcott‐Rallison syndrome in a four‐month‐old infant whose first manifestation of diabetes was diabetic ketoacidosis. Diagnosis of COVID‐19 infection after admission was considered as a possible cause of accelerated diabetic ketoacidosis (DKA) in our patient.

## 2 CASE PRESENTATION

A four‐month‐old infant boy was presented to the pediatric emergency department of Afzalipour Hospital in Kerman, Iran, with a two‐day history of fever, tachypnea, and several times vomiting. The infant was the first child and had consanguineous parents. The infant had respiratory distress, drowsiness, and severe dehydration at the time of admission. Upon admission, the infant's anthropometric measures were weight 5.5 kg, height 63 cm and the vital signs were temperature 39°C, respiratory rate 52 per minute, heart rate 180 per minute, blood pressure of 75/50 mm Hg, and oxygen saturation of 95% in room air.

**FIGURE 1:**
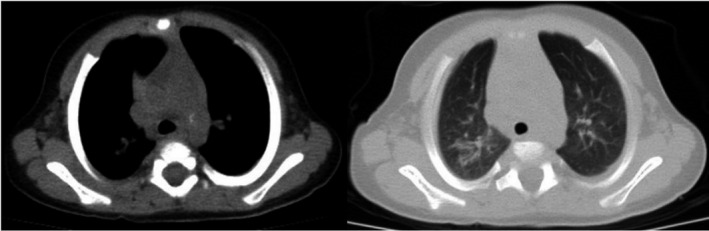
Unifocal ground‐glass opacity in the apical segment of right upper lobe (RUL) of the patient

The laboratory findings (as shown in Table 1) revealed hyperglycemia and ketoacidosis. Chest computed tomography (CT) scan reported unifocal ground‐glass opacity in the apical segment of the right upper lobe (RUL) (Figure 1). Nasopharyngeal swab for reverse transcription‐polymerase chain reaction (rRT‐PCR) test was positive for SARS‐COV‐2. The results of blood and urine cultures were negative. Parents did not report contact with any suspected febrile patient.

**TABLE 1:** Laboratory data of the patient

Investigations	Results	Reference range
Blood glucose (mg/dl)	550	70–100
Sodium (mEq/lit)	158	135–145
Potassium (mEq/lit)	5	3.5–5.5
Urea (mg/dl)	146	10–36
Creatinine (gr/dl)	0.9	0.2–0.4
AST (units/L)	148	13–35
ALT (units/L)	275	13–45
INR	1	0.86–1.2
PT (s)	12.5	11.5–15.3
PTT (s)	25	35.1–46.3
Albumin (gr/dl)	3.3	3.6–5.6
phosphorus (mg/dl)	6.5	4–6.5
Calcium (mg/dl)	4.8	4.4–5.9
CRP (mg/dl)	76.5	0–5
ESR (mm/h)	14	0–10
C‐Peptide (ng/cc)	0.7	0.9–4.2
WBC (×10^3^/µl)	4.9	4–10
RBC (×10^6^/µl)	3.4	4.5–6.3
Hb (gr/dl)	7.7	12.6 (11.1)
Hct (%)	22	36 (min:31)
Platelet (×10^9^/µl)	75	150–450
Absolute neutrophil count (×10^3^/µl)	3.724	—
Lymphocyte (×10^3^/µl)	1.078	—
Urine ketone	+++Severe	Negative
Blood culture	Negative	—
Urine culture	Negative	—
PCR assays for SARS‐COV‐2	Positive	—
Anti‐Glutamic Acid Decarboxylase Ab (IU/ml)	<0.1	<10
Anti‐islet cell Ab titer	Negative	<1/10
Anti‐Insulin Ab	0.1	<2.4
Venous blood gas (VBG)		
PH	7.01	7.35–7.45
Bicarbonate (mmol/L)	4.5	22–28
pCO_2_ (mmHg)	19.1	35–45
Base excess	−26.5	−2 to (+2)

Abbreviations: AST, aspartate aminotransferase; ALT, alanine aminotransferase; INR, international normalized ratio; PT, prothrombin time; PTT, partial thromboplastin time; CRP, C‐reactive protein; ESR, erythrocyte sedimentation rate; WBC, white blood cells; RBC, red blood cells; Hb, hemoglobin; Hct, hematocrit; PCR, polymerase chain reaction; SARS‐COV‐2, severe acute respiratory syndrome coronavirus 2; Ab, antibody.

Treatment with isotonic fluids and intravenous insulin was initiated for him and he was transferred to the pediatric intensive care unit (PICU). Regular monitoring of vital signs, electrolytes, and blood glucose was performed. Apart from supportive therapy, no other treatment was initiated for COVID‐19. Gradually, his level of consciousness and general condition improved. The DKA was resolved after two days of treatment. Therefore, breastfeeding and subcutaneous Neutral Protamine Hagedorn (NPH) insulin were initiated for the patient.

The starting dose of NPH insulin for the patient was 1.5 units at night. Blood glucose was measured regularly to adjust the insulin dose. The patient's insulin dose was gradually increased to one unit in the morning and one and a half units at night. After three days of admission, his fever subsided and after seven days of admission, he was discharged.

Results of genetic analysis of Neonatal Diabetes Mellitus (NDM) Exome Panel are shown in Table 2. A homozygous missense variant was detected in the EIF2AK3 gene. Mutations in Eukaryotic Translation Initiation Factor 2‐ALPHA Kinase 3 (EIF2AK3) on chromosome 2p11.2 can cause Wolcott‐Rallison syndrome (OMIM 226980; autosomal recessive). The patient's parents were informed about the results of the genetic test. Eventually, they were referred to genetic counseling.

**TABLE 2:** Results of genetic analysis of neonatal diabetes mellitus (NDM) exome panel

Gene (RefSeq)	Variant location	Variant	Chromosome position (GRCh37)	Zygosity	Related disease (MIM number)	Inheritance pattern	Variant classification
EIF2AK3 NM_004836	Exon 14	c.2870G>A p.Gly957Glu	Chr2: 88870507	Hom	Wolcott‐Rallison syndrome (226980)	Autosomal recessive	VUS

Abbreviations: EIF2AK3, eukaryotic translation initiation factor 2‐ALPHA kinase 3; VUS, variant of uncertain significance.

## 3 DISCUSSION

In this study, we reported WRS in a four‐month‐old infant whose first manifestation of diabetes was DKA. Most cases of diabetes that present in the first six months of life have a monogenic cause and are known as neonatal diabetes.^11^ Diabetes is a rare cause of infantile and neonatal hyperglycemia. In some cases, DKA is the first manifestation of diabetes.^12^

WRS is an autosomal recessive disease that is more common in consanguineous families.^5^ Manifestations of this disease include early‐onset diabetes, which is usually diagnosed in the first six months of life.^13^ Other manifestations of this syndrome include skeletal dysplasia, central hypothyroidism, growth retardation, and renal and hepatic dysfunction.^3^, ^4^

Homozygous mutations in the EIF2AK3 gene, located on chromosome 2p11.2 and encoding PKR‐like endoplasmic reticulum kinase (PERK), cause WRS.^5^ PERK is a transmembrane enzyme and is highly expressed in pancreatic beta cells and bone tissue.^14^ This enzyme plays a vital role in unfolded protein response (UPR) following endoplasmic reticulum stress. In case of accumulation of misfolded proteins in stress, PERK is activated.^15^, ^16^ PERK phosphorylates the alpha subunit of the eukaryotic initiation factor‐2 (EIF2A), thereby reducing the synthesis of misfolded proteins and increasing the expression of activating transcription factor 4 (ATF4), a stress‐induced transcription factor.^14^ ATF4 controls the expression of a large number of adaptive genes that allow cells to endure stress (such as hypoxia and amino acid deficiency), and in persistent stress, induces apoptosis.^17^

To the best of our knowledge, the detected variant of our patient (c.2870G>A) has not been previously reported. However, this variant can be classified as a variant of uncertain significance (VUS).

Sreeramaneni et al^18^ reported WRS in a 38 days old infant with DKA and a comatose state as the first presentation of diabetes. Asl et al^3^ study showed that from 42 patients with neonatal diabetes, pathogenic variants of EIF2AK3 were identified in 7 patients, and diagnosis of WRS was established for them.

The first presentation of WRS in our patient was DKA, and new‐onset diabetes mellitus and clinical and laboratory data did not show any other manifestation, including growth retardation and renal or liver dysfunction. This can be due to the young age of our patient, and follow‐up should be done to identify other manifestations of the disease early.

DKA is a metabolic disorder characterized by the following biochemical criteria: PH < 7.3, Serum HCO_3_ < 15, and with serum glucose greater than 200 mg/dl and the presence of concomitant ketones in the blood >3 mmol/lit or urine ketones ≥2+.^19^ Infections are common and known causes that accelerate the onset of DKA.^20^, ^21^

There are few reports of DKA as an early manifestation of type 1 diabetes caused by infection, particularly COVID‐19 infection.^22^, ^23^, ^24^ Soliman et al^23^ reported a case of DKA in an eight‐month‐old infant infected by COVID‐19. In another study, Suwanwongse et al. reported three cases of COVID‐19 newly diagnosed diabetes mellitus. In the first case, a concurrent between COVID‐19 and diabetes was observed. Although, in the other two cases, the patient was diagnosed with diabetes after the COVID‐19 infection. It is crucial to mention that all cases reported in these studies were adults.^25^ Unsworth et al study on thirty children with new‐onset diabetes mellitus between 23 months to 16.8 years old showed that 70% of them presented with DKA, and five patients had positive SARS‐CoV‐2 PCR or IgG antibody.^24^ Salmi et al study showed that admission of children to the PICU due to new‐onset type 1 diabetes mellitus increased significantly during the COVID‐19 pandemic. However, SARS‐CoV‐2 antibodies of 33 patients in their study were analyzed, and all of them were negative.^26^

The mechanism by which COVID‐19 induces DKA is unknown, but direct damage to pancreatic beta cells due to infection and acute insulin depletion can lead to DKA.^27^ Localization of ACE2 in the endocrine region of the pancreatic cells suggests that SARS‐CoV‐2 through these receptors enters the cells of the pancreas and causes cell damage.^28^

Our knowledge of COVID‐19 and diabetes is still limited. The infant was diagnosed with WRS, the first manifestation of which was diabetic ketoacidosis and concomitant with COVID‐19. Despite the severity of ketoacidosis and its association with COVID‐19, the patient responded well to the treatment and was discharged with the order of subcutaneous insulin at home. Moreover, the parents were referred to genetic counseling.

## 4 CONCLUSION

Although WRS is rare, it is known as one of the most common causes of monogenic diabetes, and the initial manifestation of permanent early‐onset diabetes mellitus in these patients can be DKA. The combination of COVID‐19 and DKA, in this case, shows that COVID‐19 can accelerate the onset of DKA and we should consider it in the clinical context. In other words, DKA as an early manifestation of diabetes at infancy can be precipitated due to COVID‐19 infection.

## CONFLICT OF INTEREST

There is no conflict of interest.

## AUTHOR CONTRIBUTIONS

EM: the endocrinologist involved in treating the patient and collaboration in writing and editing the case report. MS: the other endocrinologist was involved in treating the patient and collaboration in writing, editing, and submitting the manuscript. AB and ND: the authors of the initial draft of the manuscript and editing the article.

## ETHICS APPROVAL

The informed consent of the patient's parents has been obtained and the therapy procedure has been approved by the “Iran National Committee for Ethics in Biomedical Research” (http://ethics.research.ac.ir/IndexEn.php, no.: IR.KMU.AH.REC.1400.025).

## Data Availability

The data that support the findings of this study are available from the corresponding author upon reasonable request.
